# The neoadjuvant immunotherapy for non-metastatic mismatch repair-deficient colorectal cancer: a systematic review

**DOI:** 10.3389/fimmu.2025.1540751

**Published:** 2025-05-01

**Authors:** Hong-Xia Cui, Xiao-Quan Yang, Guang-yue Zhao, Feng-jian Wang, Xin Liu

**Affiliations:** ^1^ Department of Pharmacy, Cancer Hospital of China Medical University, Liaoning Cancer Hospital and Institute, Shenyang, Liaoning, China; ^2^ Department of General Surgery, Cancer Hospital of China Medical University, Liaoning Cancer Hospital and Institute, Shenyang, Liaoning, China; ^3^ Department of Colorectal Surgery, Cancer Hospital of China Medical University, Liaoning Cancer Hospital and Institute, Shenyang, Liaoning, China

**Keywords:** neoadjuvant immunotherapy, non-metastatic colorectal cancer, mismatch repair-deficient, pCR, meta-analysis.

## Abstract

**Background:**

Immunotherapy has become the first-line treatment for metastatic mismatch repair deficient (dMMR) colorectal cancer. The efficacy and safety of neoadjuvant immunotherapy for the treatment of non-metastatic dMMR colorectal cancer remain unclear. In this article, we explore the clinical effect and safety of neoadjuvant immunotherapy for non-metastatic dMMR colorectal cancer.

**Methods:**

We collected clinical data from the databases (PubMed, Wanfang Embase, Cochrane Library, and China National Knowledge Infrastructure databases) up to November 2024. The primary outcomes of major pathological response (MPR), pathological complete response (pCR), and other outcomes were analyzed for the final results. The secondary outcomes (pCR rates for the subgroups) were also analyzed.

**Results:**

We included 21 articles with 628 non-metastatic dMMR colorectal cancers. A pCR was found in 320/480 (66.6%) patients [effect size (ES): 0.70, 95% CI: 0.66 to 0.74] with the fixed-effects model and little heterogeneity. A MPR was found in 388/452 (85.8%) patients (ES: 0.86, 95% CI: 0.81 to 0.91) with the fixed-effects model and little heterogeneity. In the subgroup analysis, pCR rates were similar in the T1-T3 group and T4a-T4b group in the fixed-effects model with minimal heterogeneity (OR: 0.76, 95% CI: 0.48 to 1.22). The pCR rates were similar in the colon cancer group and rectal cancer group in the fixed-effects model with minimal heterogeneity (OR: 1.41, 95% CI: 0.39 to 5.12). Similar pCR rates were found in the immune monotherapy group and immune therapy plus chemotherapy group (OR: 0.74, 95% CI: 0.26 to 2.10) with the fixed-effects model and little heterogeneity.

**Conclusion:**

Neoadjuvant immunotherapy achieves high rates of pCR and MPR for non-metastatic dMMR colorectal cancer. For locally advanced T4 stage dMMR colorectal cancer, neoadjuvant immunotherapy can still achieve good pCR rates. Neoadjuvant immune monotherapy can achieve good pCRs rates, avoiding the toxic side effects caused by combined dual immunotherapy and chemo(radio)therapy. Neoadjuvant immunotherapy could be another treatment option for non-metastatic dMMR colorectal cancer.

**Systematic review registration:**

https://www.crd.york.ac.uk/prospero/, identifier CRD42024594173.

## Background

The high incidence rate of colorectal cancer (CRC) has seriously threatened human health (1). CRC can be classified into mismatch repair-deficient (dMMR) CRC and mismatch repair-proficient (pMMR) CRC based on the absence of MMR protein expression (2). Microsatellite instability (MSI) status, detected by polymerase chain reaction (PCR), is divided into microsatellite stable (MSS) and microsatellite instability-high (MSI-H). dMMR/MSI-H CRC accounted for approximately 15% of non-metastatic CRC (3, 4). Neoadjuvant therapy is an important treatment mode for CRC, especially for rectal cancer (5, 6). The FOxTROT study reported that most dMMR/MSI-H colon cancer patients had little response to neoadjuvant chemotherapy (FOLFOX or CAPOX) (7). Some relevant retrospective studies showed that dMMR/MSI-H rectal cancer had disease progression with neoadjuvant chemotherapy (fluorouracil plus oxaliplatin), but there was no disease progression in the pMMR/MSS group with neoadjuvant chemotherapy (8).

The KEYNOTE-016 study (NCT01876511) indicated that immunotherapy benefits patients with metastatic dMMR/MSI-H CRC (9). The original NICHE study cohort reported a major pathological response (MPR) rate of 95% and a pathological complete response (pCR) rate of 60% for non-metastatic dMMR/MSI-H colon cancer patients after neoadjuvant immunotherapy (10). The European Society of Oncology (ESMO) guidelines recommend pembrolizumab or nivolumab ± ipilimumab as the first-line treatment for dMMR/MSI-H metastatic CRC. Immunotherapy is superior to chemotherapy in terms of survival benefits, quality of life, and tolerability (11). Although some studies have confirmed the efficacy of neoadjuvant immunotherapy for locally advanced dMMR/MSI-H CRC, there is no relevant guideline recommending whether neoadjuvant immunotherapy can be used in non-metastatic dMMR/MSI-H CRC (12). Current evidence primarily comes from small phase II trials, which showed high pathological response rates but lacked long-term survival outcomes. Existing guidelines for non-metastatic dMMR/MSI-H CRC have lagged behind emerging evidence, reflecting gaps in evidence quality, protocol standardization, and practical implementation (2). Guidelines have hesitated to endorse neoadjuvant immunotherapy without Phase III validation.

This study focused on dMMR/MSI-H specifically due to its unique biological characteristics, including high tumor mutational burden (TMB) and increased neoantigen production, which are associated with enhanced immunogenicity and responsiveness to immune checkpoint inhibitors (13). These features make dMMR/MSI-H tumors particularly susceptible to immunotherapy, as demonstrated by higher objective response rates and durable clinical benefits in clinical trials.

Non-dMMR/MSI-H patients have a low response rate to immunotherapy (<5%). MMR/MSI status is recommended to be routinely tested (via immunohistochemistry or molecular testing) before initiating immunotherapy to identify potential beneficiaries. This testing helps avoid unnecessary toxicity and economic burden (14). Therefore, we collected relevant articles on neoadjuvant immunotherapy for non-metastatic dMMR/MSI-H CRC and tried to explain the safety and efficacy of neoadjuvant immunotherapy for non-metastatic dMMR/MSI-H CRC.

## Methods

### Literature search

This systematic review was registered on the PROSPERO website (CRD42024594173). **Supplementary Material 1** provides the details. We performed the systematic review according to the PRISMA guidelines (Preferred Reporting Items for Systematic Reviews and Meta-Analyses) (**Supplementary Material 2**).

We obtained relevant articles from various databases (Embase, PubMed, Cochrane Library, China National Knowledge Infrastructure (CNKI), and Wanfang databases). We searched the literature up to November 2024. The search terms were “colorectal cancer” and “mismatch repair-deficient” and “neoadjuvant immunotherapy” or “PD-1” or “CTLA-4”.

The PICO (population, intervention, comparator, and outcomes) model was followed to guide our literature research for the subgroup analysis. We used one of the subgroup analyses as an example. The population included non-metastatic dMMR/MSI-H CRC patients. The intervention was the colon cancer group. The comparator was the rectal cancer group. The outcome was a pCR.

### Inclusion and exclusion criteria

The inclusion criteria were as follows: (1) non-metastatic dMMR/MSI-H CRC; (2) prospective study, retrospective study, single-arm study, cohort study, and RCTs; (3) the included patients received neoadjuvant immunotherapy.

The exclusion criteria were as follows: (1) pMMR/MSS or metastatic CRC; (2) case reports, meeting, letter, and other unsuitable article types; (3) no neoadjuvant immunotherapy.

In meta-analyses, conference abstracts and case reports are excluded due to considerations of methodological rigor and result reliability. Conference abstracts typically provide only preliminary results and lack detailed study designs, methodological descriptions, and complete results. Case reports have low evidence levels, lack scientific inferential value, and carry a high risk of bias. The PRISMA guidelines explicitly recommend prioritizing the inclusion of peer-reviewed full-text publications and excluding sources with incomplete or unverified information (conference abstracts and case reports) (15).

### Data extraction and quality assessment

Two reviewers (HXC and FJW) searched the relevant studies in the literature and sorted the useful clinical data independently with the help of the revised version of MINORS (methodological index for non-randomized studies). The revised version of MINORS was used for the quality assessment of observational or non-randomized studies (16). The third reviewer (GYZ) resolved the inconsistencies between the above two authors.

**Tables 1**–**3** showed the baseline data (such as sex, country, age, and MMR status), the primary outcomes (MPR, pCR, and so on), and secondary outcomes (pCR rates for the subgroups). The details of the clinical stage and neoadjuvant immunotherapy plan are shown in **Supplementary Materials 3, 4**, respectively. No further information was obtained from the relevant authors of the included studies.

**Table 1:** Characteristics of the included articles.

Study		Country	Year		Case	Age	Sex (male/female)		ECOG (0-1)		Article type		Median time to surgery (weeks)		Lynch syndrome	
Bando et al., 2022 (19)		Japan	2022		5	58	3/2		5		PCS		12.7		NR	
Cercek et al., 2022 (20)		America	2022		16	54	6/10		16		PCS		NR		NR	
Chalabi et al., 2024 (21)		Netherlands	2024		115	60	48/67		115		PCS		NR		37	
Chen et al., 2023 (22)		China	2023		17	50	11/6		17		RCS		NR		6	
de Gooyer et al., 2024 (23)		Netherlands	2024		59	65	27/32		59		RCS		8		11	
Deng et al., 2024 (24)		China	2024		20	56	11/9		NR		RCS		NR		6	
Han et al., 2023 (25)		China	2023		12	49	7/5		NR		RCS		NR		NR	
Hu et al., H 2022 (26)		China	2022		34	49	23/11		34		PCS		NR		NR	
Kothari et al., 2022 (27)		America	2022		9	55.9	5/4		NR		RCS		7		3	
Li et al., 2023 (28)		China	2023		26	NR	NR		26		RCS		NR		NR	
Li et al., 2024 (29)		China	2024		24	49	5/19		20		RCS		NR		12	
Liu et al., 2024 (30)		China	2022		26	NR	NR		26		RCS		NR		NR	
Liu et al., 2022 (31)		China	2022		26	NR	NR		26		RCS		NR		NR	
Ludford et al., 2023 (32)		America	2023		27	NR	NR		NR		PCS		NR		NR	
Pan et al., 2024 (33)		China	2024		11	44.6	3/8		11		RCS		NR		NR	
Pei et al., 2023 (34)		China	2023		11	59	7/4		NR		RCS		NR		NR	
Xiao et al., 2023 (35)		China	2023		73	48	44/29		NR		RCS		16		27	
Xie et al., 2023 (36)		China	2023		13	53	5/8		NR		PCS		2		NR	
Yang et al., 2023 (37)		China	2023		20	55	13/7		20		RCS		NR		NR	
Yu et al., 2024 (38)		China	2024		52	54.5	23/29		52		RCS		5.3		20	
Zhang et al., 2022 (39)		China	2022		32	44	17/15		32		RCS		NR		NR	
Study	MMR status			Immunotherapy drugs				Other therapy		Tumor location						
										multiple		right	transverse	left		
														colon		rectum
Bando et al., 2022 (19)	NR			Nivolumab				CRT				0	0	0		5
Cercek et al., 2022 (20)	MSH2 and MSH6(6), MLH1 and PMS2(3), MSH2(3), MSH6(2), PMS2(2)			Dostarlimab				NR				NR	NR	NR		16
Chalabi et al., M 2024 (21)	NR			Nivolumab				NR				78	17	20		0
Chen et al., 2023 (22)	MSH2 or MSH6, or both (9); MLH1 or PMS2, or both (7)			Sintilimab				CapeOX				0	0	0		17
de Gooyer et al., 2024 (23)	NR			Nivolumab and relatlimab				NR				48	6	NR		NR
Deng et al., 2024 (24)	MLH1, MSH2 and PMS2(2), MSH2 and MSH6(4), MLH1 and PMS2(7), MSH2(4), PMS2(2)			Nivolumab, ipilimumab, pembrolizumab, sintilimab, and tislelizumab				CapeOX, FOLFIRI, radiotherapy		2		NR	NR	NR		5
Han et al., 2023 (25)	MLH1(5), MSH2(3), MSH6(3), PMS2(5)			Permbrolizumab, nivolumab, and				NR				5	2	NR		NR
Hu et al., 2022 (26)	MSH2 or MSH6, or both (20); MLH1 or PMS2, or both (16)			toripalimab				NR				17	6	7		6
Kothari et al., 2022 (27)	NR			Permbrolizumab and nivolumab				chemotherpay				6	0	2		1
Li et al., 2023 (28)	NR			Permbrolizumab and sintilimab				CapeOX				NR	NR	NR		7
Li et al., 2024 (29)	NR			Pembrolizumab, sintilimab, toripalimab, and camrelizumab				CAPOX				8	3	5		8
Liu et al., 2024 (30)	NR			Pembrolizumab, ipilimumab, and nivolumab				FOLFOX XELOX				NR	NR	NR		2
Liu et al., 2022 (31)	NR			PD-1 inhibitor, and CTLA-4 inhibitor				CapeOX				NR	NR	NR		13
Ludford et al., 2023 (32)	NR			Pembrolizumab,				NR				NR	NR	NR		8
Pan et al., 2024 (33)	MSH2 or MSH6, or both (3); MLH1 or PMS2, or both (8)			nivolumab, and ipilimumab				NR				NR	NR	NR		NR
Pei et al., 2023 (34)	MSH2 and MSH6(3), MLH1 and PMS2(5), MSH2(1), PMS2(2)			Sintilimab				NR				3	5	2		1
Xiao et al., 2023 (35)	NR			Toripalimab, pembrolizumab, nivolumab, sintilimab, camrelizumab, and tislelizumab				XELOX FOLFOXIRI		7		18	14	16		18
Xie et al., 2023 (36)	NR			Pembrolizumab, toripalimab, and sintilimab				mFOLFOX6				NR	NR	NR		13
Yang et al., 2023 (37)	MLH1, MSH2 and PMS2(1), MSH2 and MSH6(3), MLH1 and PMS2(4), MSH2(2), MSH2(3),			Pembrolizumab, sintilimab, and tislelizumab.				NR		0		0	0	0		20
Yu et al., 2024 (38)	NR			Camrelizumab and apatinib				NR		6		18	12	10		6
Zhang et al., 2022 (39)	NR			Pembrolizumab, sintilimab, and tiselizumab				NR				11	4	9		8

dMMR, mismatch repair-deficient; MSI-H, microsatellite instability-high; pCR. pathological complete response; MPR. major pathological response; ORR. objective response rate; cCR. complete clinical response; CRT. chemoradiotherapy; irAEs. immune-related adverse events; RCS. retrospective clinical study; PCS, prospective clinical study; NR, no record.

The order of additional information was range, standard deviation, percentage, or NR (if not reported).

**Table 2:** Primary outcomes.

Study	MPR (%)	pCR (%)	Postoperative complications (%)	irAEs (%)	T4-PCR (%)	ORR (%)	cCR (%)
Bando et al., 2022 (19)	3 (60)	3 (60)	NR	NR	NR	3 (60)	NR
Cercek et al., 2022 (20)	NR	NR	NR	NR	NR	NR	12 (100)
Chalabi et al., 2024 (21)	105 (91.3)	75 (65.2)	22 (11.9)	73 (63.4)	46 (63.8)	109 (98.1)	NR
Chen et al., 2023 (22)	5 (83.3)	3 (50)	NR	9 (53)	2 (66.7)	15 (93.7)	9 (56.2)
de Gooyer et al., 2024 (23)	54 (92)	40 (68)	22 (37)	47 (80)	26 (65)	NR	NR
Deng et al., 2024 (24)	NR	12 (70.6)	NR	NR	8 (80)	15 (88.2)	NR
Han et al., 2023 (25)	7 (70)	7 (70)	NR	7 (58.8)	NR	7 (70)	NR
Hu et al., 2022 (26)	29 (85.3)	26 (76.4)	NR	20 (58.8)	24 (80)	NR	NR
Kothari et al., 2022 (27)	9 (100)	8 (88.8)	NR	NR	5 (83.3)	5 (55.5)	NR
Li et al., 2023 (28)	NR	10 (52.6)	NR	NR	NR	NR	3 (15.7)
Li et al., 2024 (29)	8 (53.3)	7 (46.7)	NR	NR	6 (60)	NR	2 (11.7)
Liu et al., 2024 (1)	2 (66.7)	2 (66.7)	NR	NR	NR	4 (100)	1 (25)
Liu et al., 2022 (31)	17 (65.4)	15 (57.6)	NR	NR	NR	NR	NR
Ludford et al., 2023 (32)	NR	11 (78.5)	NR	NR	NR	NR	NR
Pan et al., 2024 (33)	8 (80)	8 (80)	NR	NR	2 (100)	NR	NR
Pei et al., 2023 (34)	11 (100)	10 (90.9)	0	NR	4 (80)	8 (72.7)	NR
Xiao et al., 2023 (35)	31 (63.2)	28 (59.1)	4 (8)	10 (13.7)	22 (59.5)	62 (84.9)	17 (23.3)
Xie et al., Y 2023 (36)	12 (92.3)	11 (84.6)	NR	NR	NR	9 (69.2)	NR
Yang et al., 2023 (37)	13 (100)	11 (84.6)	3 (23.1)	8 (40)	8 (88.9)	13 (100)	7 (35)
Yu et al., 2024 (38)	48 (92.3)	14 (61)	NR	52 (98)	NR	NR	28 (54)
Zhang et al., X 2022 (39)	25 (86.2)	22 (75.9)	3 (10.3)	NR	NR	29 (100)	3 (9.4)
Total	388 (85.8)	320 (66.6)	51 (19.3)	226 (59)	153 (68.3)	279 (89.7)	82 (33.8)

dMMR, mismatch repair-deficient; MSI-H, microsatellite instability-high; pCR, pathological complete response; MPR, major pathological response; ORR, objective response rate; cCR, complete clinical response; CRT, chemoradiotherapy; irAEs, immune-related adverse events; RCS, retrospective clinical study; PCS, prospective clinical study, NR, no record.

The order of additional information was range, standard deviation, percentage, or NR (if not reported).

**Table 3:** Secondary outcomes.

Study	pCR (%)		pCR (%)		pCR (%)	
	T1-T3	T4a-T4b	Colon	Rectum	Immune monotherapy	Immune therapy plus chemotherapy
Bando et al., 2022 (19)	NR	NR	NR	NR	NR	NR
Cercek et al., 2022 (20)	NR	NR	NR	NR	NR	NR
Chalabi et al., 2024 (21)	29 (67.4)	46 (63.9)	NR	NR	NR	NR
Chen et al., 2023 (22)	1 (33.3)	2 (66.7)	NR	NR	NR	NR
de Gooyer et al., 2024 (23)	14 (74)	26 (65)	NR	NR	NR	NR
Deng et al., 2024 (24)	4 (57.1)	8 (80)	NR	NR	8 (61.5)	4 (100)
Han et al., 2023 (25)	NR	NR	NR	NR	NR	NR
Hu et al., 2022 (26)	2 (50)	24 (80)	23 (79.3)	5 (83.3)	NR	NR
Kothari et al., 2022 (27)	3 (100)	5 (83.3)	7 (87.5)	1 (100)	5 (100)	3 (75)
Li et al., 2023 (28)	4 (40)	6 (66.7)	8 (57.1)	2 (40)	7 (43.7)	3 (100)
Li et al., 2024 (29)	1 (20)	6 (60)	NR	NR	6 (54.5)	1 (25)
Liu et al., 2024 (1)	NR	NR	NR	NR	1(50)	1 (100)
Liu et al., 2022 (31)	NR	NR	NR	NR	NR	NR
Ludford et al., 2023 (32)	NR	NR	NR	NR	NR	NR
Pan et al., 2024 (33)	2 (25)	2 (100)	NR	NR	NR	NR
Pei et al., 2023 (34)	6 (100)	4 (80)	9 (90)	1 (100)	NR	NR
Xiao et al., 2023 (35)	6 (50)	22 (59.5)	NR	NR	NR	NR
Xie et al., 2023 (36)	NR	NR	NR	NR	7 (87.5)	4 (80)
Yang et al., 2023 (37)	3 (75)	8 (88.9)	NR	NR	NR	NR
Yu et al., 2024 (38)	NR	NR	NR	NR	NR	NR
Zhang et al., 2022 (39)	NR	NR	NR	NR	NR	NR
Total	75 (60.5)	159 (68.2)	47 (77)	9 (69.2)	34 (61.8)	16 (76.2)

dMMR, mismatch repair-deficient; MSI-H, microsatellite instability-high; pCR, pathological complete response; MPR, major pathological response; ORR, objective response rate; cCR, complete clinical response; CRT, chemoradiotherapy; irAEs, immune-related adverse events; RCS, retrospective clinical study; PCS, prospective clinical study; NR, no record.

The order of additional information was range, standard deviation, percentage, or NR (if not reported).

### Statistical analysis

We used STATA software to perform statistical analysis on single-group binary variables. The effect size (ES) in the results typically reflected the overall effect derived from specific statistical approaches, such as Logit transformation or Freeman–Tukey double arcsine transformation (17). For the statistical analysis of two-group binary variables (colon vs. rectal cancer group), we utilized the risk ratio {RR=[a/(a+b)]/[c/(c+d)]} or odds ratio (OR=a*d/b*c) to compare the relative risks of event occurrence between the two groups using RevMan software. A random effects model and fixed effects model were used to analyze the data with high heterogeneity (*I* ^2^≧50%) and low heterogeneity (*I* ^2^<50%), respectively. The fixed-effects model had high statistical power, with narrow confidence intervals, making the results more “significant” and suitable for idealized scenarios. However, in studies with high heterogeneity, it could lead to false-positive results. In contrast, the random-effects model produced more conservative statistical results, which were more robust and better suited for heterogeneous scenarios. Nevertheless, it had lower statistical power and was sensitive to small sample sizes, making it more aligned with real-world situations (18). Publication bias was assessed using funnel plots.

## Results

### Study selection

**Figure 1:**
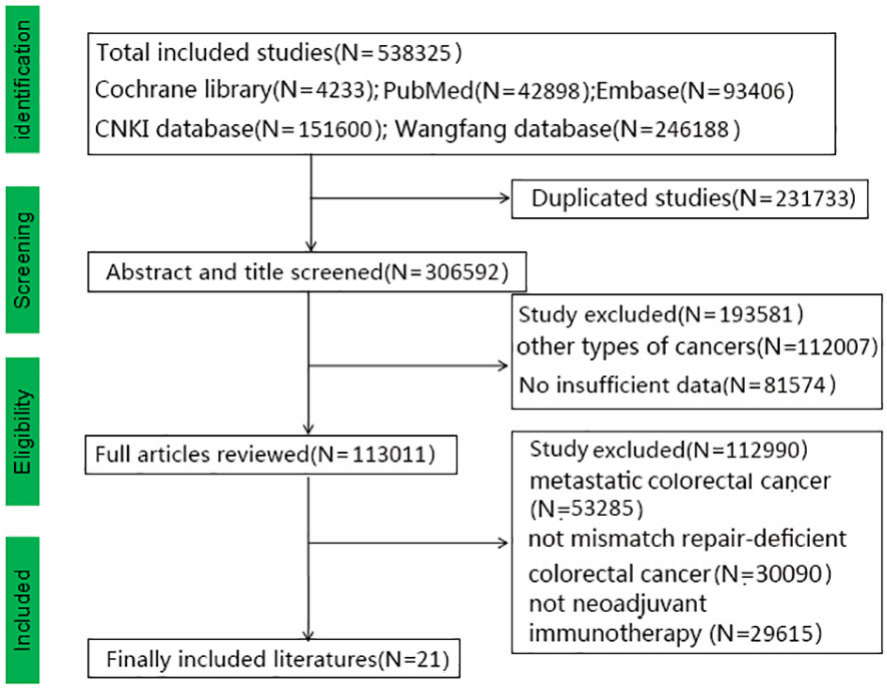
Study selection followed by PRISMA diagram.

We obtained 538,325 relevant studies from the medical databases. According to the inclusion and exclusion criteria, we excluded studies on metastatic colorectal cancer (N=53,285) and those that did not include mismatch repair-deficient colorectal cancer (N=30,090) and neoadjuvant immunotherapy (N=29,615) (**Figure 1**). In total, 21 articles with 628 patients with non-metastatic dMMR colorectal cancer were collected, including 474 (75.5%) with colon cancer and 154 (24.5%) with rectal cancers (19–39). **Table 1** shows the baseline data of the included studies. Eight studies reported MMR status. Alterations in MSH2 were most common while pathogenic alterations in MSH6, MLH1, and PMS2 were also present. There were 15 retrospective studies (13 Eastern studies and 2 Western studies) and 6 prospective studies (3 Eastern studies and 3 Western studies). All the patients had an Eastern Cooperative Oncology Group Performance Status (ECOG) score of 0-1. All the included studies achieved 12–14 points with high-moderate quality according to MINORS standard and the specific information is provided in **Supplementary Material 5**. Chalabi et al. (2024) and Chalabi et al. (2022) collected the same patients (the first patient was enrolled in March 2017), therefore this study did not include Chalabi et al. (2022) (10, 21). Li et al. (2023) included colorectal cancer, duodenal cancer, gastric cancer, and colorectal liver metastases (28). We selected the eligible patients according to the criteria, which could result in some missing data (such as postoperative complications, MRP, and others). Pembrolizumab, nivolumab, and sintilimab were the most used immunotherapy drugs. Other immunotherapy drugs, such as dostarlimab, toripalimab, camrelizumab, tiselizumab, and others, were also used for colorectal cancer. Bando (2022) used neoadjuvant immunotherapy combined with chemoradiotherapy (capecitabine and radiation to a dose of 50.4 Gy), while neoadjuvant immunotherapy combined with chemotherapy (CapeOX or FOLFOXIRI) was used in some studies (Kothari, 2022; Xiao, 2023; Liu, 2022; Li, 2023) (19, 27, 28, 31, 35).

According to the RECIST criteria, the objective response rate (ORR) was 89.7% after neoadjuvant immunotherapy. A pCR and cCR were observed in 320 (66.6%) and 82 (33.8%) patients after neoadjuvant immunotherapy, respectively. **Table 2** shows the information on the primary outcomes [pCR, MPR, postoperative complications, immune-related adverse events (irAEs), T4-pCR, and ORR]. **Table 3** shows the information on the secondary outcomes, i.e., pCR rates between different subgroups.

### Primary outcomes: pCR, MPR, postoperative complications, immune-related adverse events, T4-pCR, and ORR

**Figure 2:**
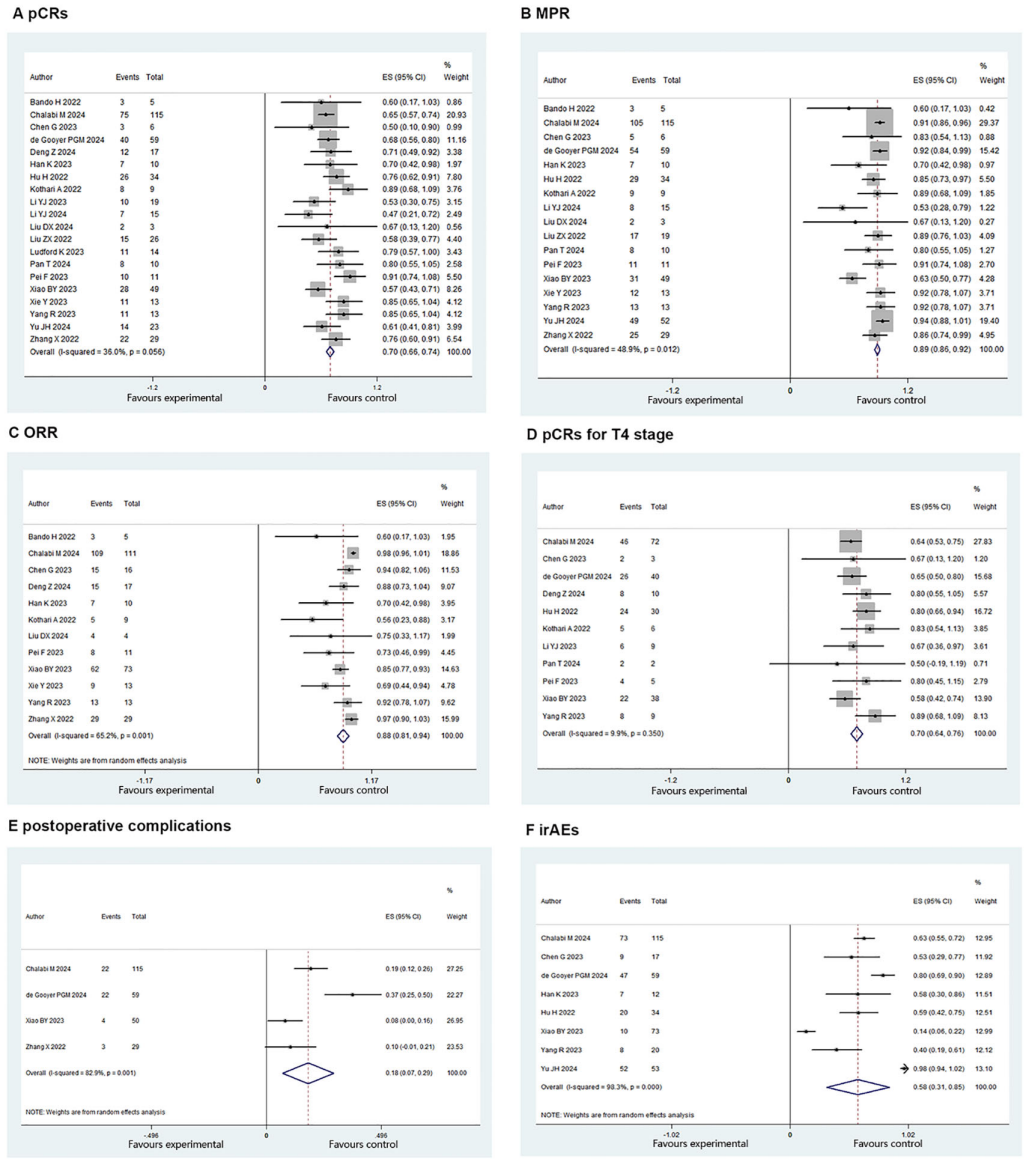
Primary outcomes of neoadjuvant immunotherapy for non-metastatic MMR colorectal cancer. **(A)** pathological complete response (PCRS) **(B)** major pathological response(MPR): **(C)** objective response rate (ORR): **(D)** PCRe for 14 stage : **(E)** postoperative **(F)** immune-related adverse events (irAEs).

The pCR rate was reported by 20 studies, and 320/480 (66.6%) patients had a pCR (ES: 0.70, 95% CI: 0.66 to 0.74, *P*<0.01, chi^2^ = 29.69, *P*>0.05, *I* ^2^ = 36%, **Figure 2**) and there was low heterogeneity, thus the fixed-effects model was used. MPR was reported by 17 studies. An MPR was found in 388/452 (85.8%) patients (ES: 0.86, 95% CI: 0.81 to 0.91, *P*<0.01, chi^2^ = 31.31, *P*<0.05, *I* ^2^ = 48.9%, **Figure 2**), and there was low heterogeneity, thus, the fixed-effects model was used.

In total, 51 cases of postoperative complications occurred. The postoperative complications rate was estimated at 19.3% (ES: 0.18, 95% CI: 0.07 to 0.29, *P*<0.01, chi^2^ = 17.50, *P*<0.01, *I* ^2^ = 82.9%, **Figure 2**) with the random-effects model and there was high heterogeneity. The irAE rate was estimated at 59% (226/383) (ES: 0.58, 95% CI: 0.31 to 0.85, *P*<0.01, chi^2^ = 404.9, *P*<0.01, *I* ^2^ = 98.3%, **Figure 2**) with the random-effects model and there was high heterogeneity.

The T4-pCR rate was reported in 11 studies. A T4-pCR was found in 153/224 (68.3%) patients (ES: 0.70, 95% CI: 0.64 to 0.76, *P*<0.01, chi^2^ = 11.1, *P*=0.35, *I* ^2^ = 9.9%, **Figure 2**) with the fixed-effects model and there was low heterogeneity. ORR was reported by 12 studies and it was found in 279/311 (89.7%) patients (ES: 0.88, 95% CI: 0.81 to 0.94, *P*<0.01, chi^2^ = 31.6, *P*<0.01, *I* ^2^ = 65.2%, **Figure 2**) with the random-effects model. There was high heterogeneity.

### Secondary outcomes (subgroup analysis): pCR for T1-T3 vs. T4a-T4b, pCR for colon cancer vs. rectal cancer, and pCR for immune monotherapy vs. immunetherapy plus chemotherapy

**Figure 3:**
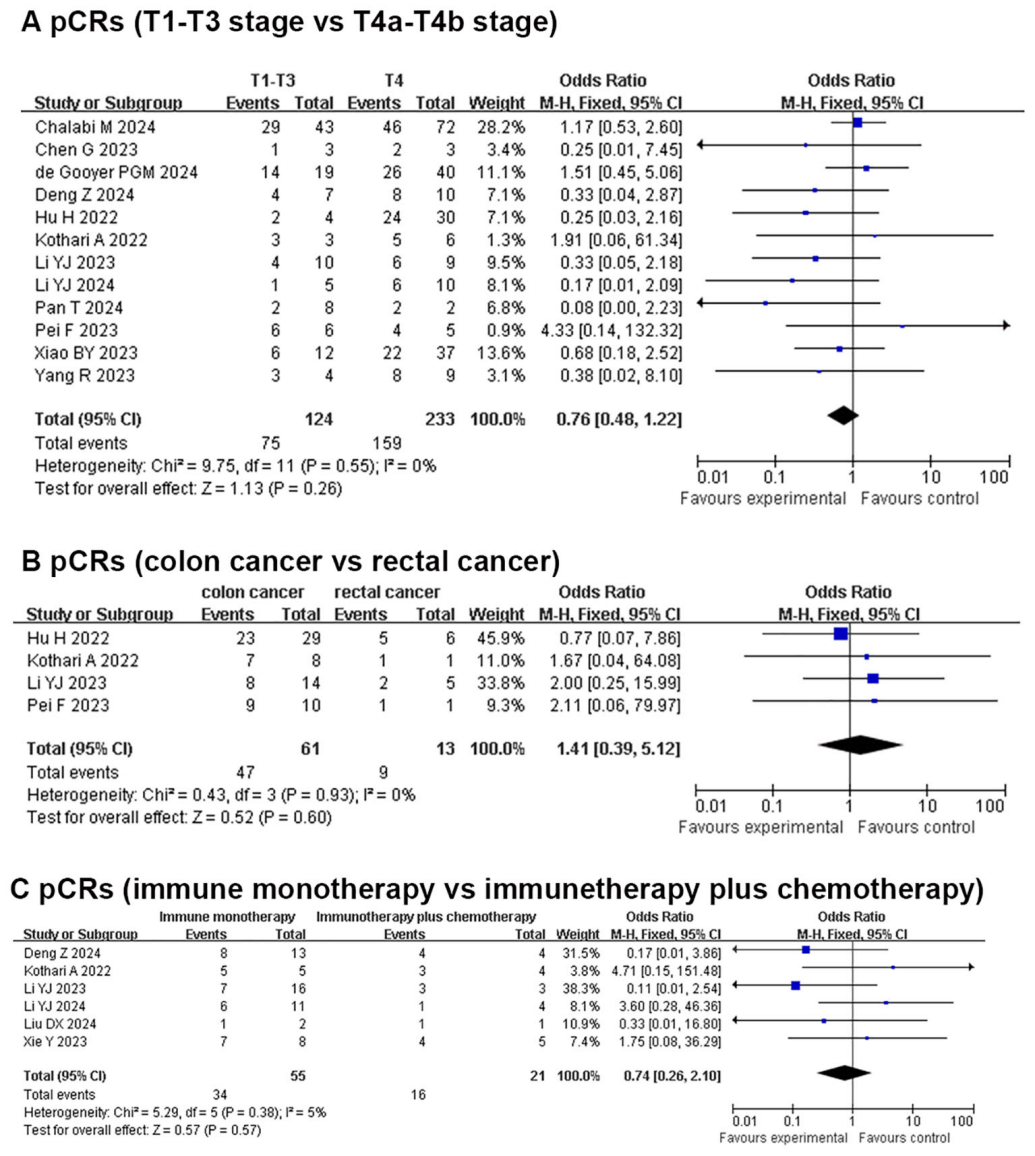
Secondary outcomes of neoadjuvant immunotherapy for non-metastatic dMMR colorectal cancer. **(A)** PCRs (T1-T3 stage vs T4a-T4b stage); **(B)** PCRs (colon cancer vs rectal cancer); **(C)** PCRs (immune monotherapy vs immunetherapy plus chemotherapy).

In total, 12 studies reported the clinical data of pCR for a T1-T3 group and a T4a-T4b group. The pCR rate was similar in the two groups in the fixed-effects model with minimal heterogeneity (OR: 0.76, 95% CI: 0.48 to 1.22, *P*=0.26, chi^2^ = 9.75, *P*=0.55, *I* ^2^ = 0%, **Figure 3**). Four studies reported the clinical data of pCR for the colon cancer group and rectal cancer group. The pCR rate was similar in the two groups in the fixed-effects model with minimal heterogeneity (OR: 1.41, 95% CI: 0.39 to 5.12, *P*=0.6, chi^2^ = 0.43, *P*=0.93, *I* ^2^ = 0%, **Figure 3**). Six studies reported the clinical data of pCR for an immune monotherapy group and an immune therapy plus chemotherapy group. The pCR rate was similar in the two groups in the fixed-effects model with little heterogeneity (OR: 0.74, 95% CI: 0.26 to 2.10, *P*=0.57, chi^2^ = 5.29, *P*=0.38, *I* ^2^ = 5%, **Figure 3**).

### Publication bias

The publication bias was visualized using RevMan 5.0 software with the clinical data of pCR. We found that the points were evenly distributed in the forest plot.

## Discussion

The DNA mismatch repair system is a testing system composed of multiple interacting proteins. The system recognizes and repairs base mismatches that occur during DNA replication after activation. The loss of function of any protein in MMR can cause defects in the MMR system, leading to the accumulation of microsatellite errors and making it susceptible to being attacked by the immune system (40, 41). Therefore, immunotherapy can mobilize immune cells to effectively kill tumor cells in dMMR/MSI-H tumors. The KEYNOTE-177 study demonstrated that immunotherapy improves survival time and ORR for metastatic dMMR/MSI-H CRC. The National Comprehensive Cancer Network (NCCN) guidelines recommend pembrolizumab as the first-line treatment of metastatic/unresectable dMMR/MSI-H CRC (Category 1 recommendation) (42).

At present, for some malignant tumors (such as melanoma, urothelial carcinoma, and other tumors), immunotherapy is not only applied in the first-line treatment for metastatic tumors but has also achieved significant clinical results in neoadjuvant immunotherapy (43, 44). A clinical case report reported two patients with locally advanced rectal cancer with dMMR/MSI-H status who received neoadjuvant immunotherapy (nivolumab) and achieved a complete response (CR) (45). The NICHE study showed that all the patients with dMMR/MSI-H colon cancer responded to neoadjuvant nivolumab plus ipilimumab with a high pCR rate (46). As the efficacy and safety of neoadjuvant immunotherapy for non-metastatic dMMR/MSI-H CRC has not been fully elucidated, we tried to use the available clinical data to explain the safety and clinical effects of neoadjuvant immunotherapy for non-metastatic dMMR/MSI-H CRC.

### Outcome results

#### pCR, MPR, postoperative complications, immune-related adverse events, T4-pCR, and ORR

The pCR rate for neoadjuvant chemo(radio)therapy for CRC was approximately 5%–15%. The pCR rate for neoadjuvant immunotherapy was 36% for non-metastatic CRC, while the pCR rate for neoadjuvant immunotherapy was 59.6% for non-metastatic dMMR/MSI-H CRC (47, 48). In our study, the pCR rate for neoadjuvant immunotherapy was 66.9% for non-metastatic dMMR/MSI-H CRC. Especially with the addition of the NICHE-2 study, the increased clinical sample sizes made the results more convincing. In Rahma et al.’s study, it was further confirmed that neoadjuvant immunotherapy had higher pCR and R0 resection rates than neoadjuvant chemoradiotherapy (49).

Loria et al. found that 50% to 91% of non-metastatic dMMR CRC with neoadjuvant immunotherapy could achieve ypT0N0 pathology (50). In our previous research, we further confirmed the excellent clinical efficacy of neoadjuvant immunotherapy (51). The pCR rate for neoadjuvant immunotherapy for locally advanced T4 stage dMMR/MSI-H CRC was 68.2%, which was exciting news for partially locally advanced T4b tumors (52). For locally advanced T4b tumors with dMMR/MSI-H status, the NCCN recommends neoadjuvant immunotherapy, while the short courses of neoadjuvant immune checkpoint inhibition will continue to expand with more ongoing clinical trials. The MPR and ORR rates were 85.8% and 89.7%, respectively, in our study. Compared with the clinical statistics of neoadjuvant chemo(radio)therapy, MPR and ORR were significantly improved. These data reflected the effectiveness of neoadjuvant immunotherapy at the pathological and imaging levels. For locally advanced T4b tumors with dMMR/MSI-H status, neoadjuvant immunotherapy can cause tumor downgrading and transform unresectable T4b tumors into resectable T1-T4a tumors, which is beneficial for complete resection and achieving curative effects.

### Adverse events and postoperative complications

The most common irAEs were endocrine disorders, skin disease, gastrointestinal reactions, and others (53). The incidence of irAEs was 59% in our study, similar to the incidence of irAEs reported in other studies (54). Most irAEs were grade I-II with mild clinical reactions or laboratory abnormalities and could be treated symptomatically. The incidence of severe irAEs was very low, including hepatic damage, neurological damage, cardiac toxicity, and others. Severe irAEs required immediate discontinuation and timely treatment. Therefore, choosing a suitable immunotherapy drug, dosage, and administration time could effectively avoid the occurrence of irAEs.

The incidence of postoperative complications was 19.3%, and most postoperative complications were abdominal infection and anastomotic leakage (grade I-II). Chalabi et al.’s (2024) study reported grade III postoperative complications with an incidence rate of 10%. Most patients with postoperative complications were discharged smoothly with conservative symptomatic treatment (55).

One multicenter retrospective study reported that 76 patients with locally advanced rectal cancer were given 45 Gy for pelvis tumors, and 52.5 to 57.5 Gy for local tumors. The pCR rate was 27.8%, the incidence of surgical complications was 18.1%, and the incidence of grade 3-4 adverse reactions was 10.5% (56). Immunotherapy was different from chemo(radio)therapy and did not increase tissue edema and fibrosis, which was beneficial for surgery. Based on the above results, we speculate that neoadjuvant immunotherapy did not significantly increase postoperative complications, but more studies are still needed to confirm this.

### Subgroup analysis: pCR for T1-T3 vs. T4a-T4b, pCR for colon cancer vs. rectal cancer, and pCR for immune monotherapy vs. immunetherapy plus chemotherapy

The pCR rate was similar in the T1-T3 group (60.5%) and the T4a-T4b group (68.2%) after neoadjuvant immunotherapy without statistical difference. For locally advanced T4b patients with dMMR/MSI-H status, neoadjuvant immunotherapy was beneficial for tumor progression and achieving curative resection (57). The MRP and pCR rates were 85.4% and 59.5% in T4b tumor with neoadjuvant immunotherapy, as reported by Xiao et al. (2023), with an acceptable safety profile and a low recurrence rate (35). The incidence of irAEs and surgical complications was similar to other studies. Therefore, we speculate that neoadjuvant immunotherapy could also preserve organs, reduce surgical trauma, and avoid combined organ resection, especially for non-metastatic unresectable T4b CRC with dMMR/MSI-H status.

The rectum is located in the pelvic cavity, with an embryonic origin in the hindgut, and its anatomical structure is complex (proximity to the anal sphincter and rich neurovascular supply), making surgery and radiotherapy challenging. Some patients require a permanent colostomy due to low tumor location necessitating anal resection. In contrast, a right hemicolectomy (originating from the embryonic midgut) and left hemicolectomy (originating from the hindgut) were associated with relatively lower surgical difficulty, did not involve permanent colostomy, and caused less trauma to patients (58). The proportion of MSI-H was higher in right-sided colon cancer (approximately 15%–20%) compared to rectal cancer (approximately 5%–10%). In our study, the pCR rate was similar in colon cancer (77%) and rectal cancer (69.2%) after neoadjuvant immunotherapy, and there was no statistical difference between the two groups. The pCR rate for neoadjuvant immunotherapy was much higher than the pCR rate for neoadjuvant chemo(radio)therapy. High pCRs rate of neoadjuvant immunotherapy could improve the rectal sphincter preservation rate without surgery, especially for low advanced rectal cancer (59). It could eliminate the trauma caused by surgery, improve the quality of life and avoid adverse events such as anterior resection syndrome and abdominal infections. In addition, watch and wait (W&W) strategy may be safe for rectal cancer patients with cCR status after neoadjuvant immunotherapy, anal examination, colonoscopy and MRI were need to completed regularly (60).

In our study, the pCR rate was similar in the immune monotherapy group (61.8%) and immune therapy plus chemotherapy group (76.2%). Yang et al. (2023) reported the efficacy of PD-1 monotherapy for locally advanced rectal cancer with dMMR/MSI-H status and the pCR and MRP rates were 84.6% and 100%, respectively (37). Li et al. (2024) pointed out that neoadjuvant immune monotherapy could achieve a high tumor complete response in initially resected difficult dMMR/MSI-H CRC. Neoadjuvant immune monotherapy could achieve cCR status, even for stage IV colorectal cancer (29). The KEYNOTE-177 trial demonstrated that pembrolizumab monotherapy as first-line treatment for dMMR/MSI-H metastatic CRC achieved a median progression-free survival (PFS) of 16.5 months (vs. 8.2 months with chemotherapy) and a 3-year overall survival rate of 55%vs. 48.6%. The incidence of grade 3-4 immune-related adverse events (irAEs) was approximately 14-20%,primarily including colitis (8%) and hepatitis (5%) (61). The treatment was well-tolerated and particularly suitable for elderly patients (>70 years) or those with complications. Immune monotherapy is considered a highly effective and low-toxicity first-line option.

In contrast, combination immunotherapy aimed for higher response rates at the cost of increased toxicity. The CheckMate-142 trial showed that nivolumab plus ipilimumab in metastatic dMMR/MSI-H CRC resulted in an ORR of 69%, a CR rate of 13%, and durable responses. However, the incidence of grade 3-4 irAEs was 32% (12% colitis, 10% hepatitis), with a significantly increased need for hospitalization or immunosuppressive interventions (62). The KEYNOTE-651 trial indicated that pembrolizumab combined with FOLFOX chemotherapy as a first-line treatment for MSS CRC achieved an ORR of 45% but with a notable increase in grade 3-4 neutropenia (50%) and diarrhea (15%). Local radiotherapy combined with PD-1 inhibitors can activate the “abscopal effect,” but the risk of radiation enteritis (10%–20%) combined with immune-related colitis was a concern (63). Based on the above results, we speculated that patients with non-metastatic dMMR/MSI-H CRC could also benefit greatly from immune monotherapy. Immune monotherapy was the preferred first-line option due to its high efficacy and low toxicity, while combination therapy was reserved for high-risk patients requiring rapid symptom relief.

### Novelty of the study and outlook for RCTs

We attempted to use the latest research to explore the clinical efficacy of neoadjuvant immunotherapy for non-metastatic dMMR/MSI-H CRC. Especially with the addition of the NICHE-2 study, the clinical data have become larger than ever before. We explored many observation indicators (pCR, MPR, postoperative complications, irAEs, and others) to clarify the advantages and disadvantages of neoadjuvant immunotherapy for non-metastatic dMMR/MSI-H CRC. We conducted a subgroup analysis and found that patients with T4 stage non-metastatic dMMR/MSI-H CRC can achieve a high pCR rate, and immune monotherapy could achieve good clinical efficacy.

Existing clinical trials focused primarily on monotherapy or combination therapy (PD-1 and CTLA-4). We expect more multi-arm randomized controlled trials (two-arm or three-arm designs) will clarify the advantages and disadvantages of different combination regimens (dual immunotherapy and immunotherapy combined with chemotherapy or targeted therapy) and define the efficacy-toxicity balance of these regimens. Long-term follow-up could reveal the curative potential and resistance mechanisms of immunotherapy (64). Since some dMMR/MSI-H patients did not respond well to immunotherapy, more refined stratification biomarkers are needed to enable personalized treatment based on multi-omics biomarkers.

### Neoadjuvant immunotherapy plan, prognosis, and resistance mechanisms

PD-L1, PD-1, or CTLA-4 monoclonal antibodies can block tumor immune escape and restore the anticancer function of the autoimmune system (65). PD-1 blockade (pembrolizumab, nivolumab, sintilimab, toripalimab, camrelizumab, and others) and CTLA-4 blockade (ipilimumab and others) have been applied in immunotherapy for colorectal cancer, while the application of PD1 blockade is more widespread than CTLA-4 blockade. Many studies recommend using PD1 for 4-6 cycles, such as pembrolizumab (200mg every 3 weeks), nivolumab (240 mg every 2 weeks), dostarlimab (500 mg every 3 weeks), and others. The next treatment plan will then be determined based on relevant examinations. Chalabi et al. (2024) reported that PD-1 (two nivolumab 3 mg/kg) combined with CTLA-4 (single ipilimumab 1 mg/kg) blockade achieved a pCR rate of 68% and an MRP rate of 95% (21). At the same time, the incidence of irAEs was 63%, which was higher than that of the monotherapy group. Neoadjuvant immune monotherapy can reduce the irAEs of dual immunotherapy and chemo(radio)therapy. The results of ongoing clinical research could ascertain the direction of the immunotherapy treatment mode and a suitable population. There was no consensus on the clinical treatment of neoadjuvant immunotherapy combined with neoadjuvant radiotherapy (SCRT or IMRT, 25-50.4 Gy) and chemotherapy (FOLFOX or CAPOX) for non-metastatic dMMR/MSI-H CRC. Unlike pMMR/MSS CRC, neoadjuvant immunotherapy can achieve an effective clinical treatment effect for non-metastatic dMMR/MSI-H CRC. The selection of immunotherapy drugs, treatment cycles, and treatment plans was still inconclusive, and further research is needed to explore this.

Traditional chemotherapy agents (such as oxaliplatin and fluorouracil) were relatively low in cost and the drugs were covered under national medical insurance reimbursement programs with single-cycle costs typically ranging from ¥ 3,000- ¥ 5,000. However, chemotherapy required multiple treatment cycles (usually 4–6 cycles), and the total cost increased significantly when combined with expenses for imaging monitoring and side-effect management (bone marrow suppression and gastrointestinal reactions). In contrast, PD-1/PD-L1 inhibitors (such as pembrolizumab and camrelizumab) had higher per-cycle costs (approximately ¥15,000–¥30,000) and most were not fully covered by insurance, resulting in a higher out-of-pocket burden for patients. Nevertheless, immunotherapy required shorter treatment durations (typically 2–4 cycles) and it demonstrated rapid tumor regression for dMMR/MSI-H CRC, which reduced the complexity of subsequent surgery. Hence, neoadjuvant immunotherapy was potentially more cost-effective for dMMR/MSI-H CRC. Despite higher upfront drug costs, the pCRs and MRP rate was significantly superior to chemotherapy. It could reduce surgical difficulty and the risk of postoperative complications, thereby reducing long-term medical expenses.

Due to neoadjuvant immunotherapy being in the exploratory stage, there was no specific consensus on drug regimens, cycles, and dosages. At this time, PD-1, PD-L1, and CTLA-4 inhibitors are commonly used in clinical treatment, but the endpoints of the studies were similar, including MRP, PCR, ORR, OS, and other indicators. Therefore, we extracted common efficacy endpoints (ORR, MRP, PCR, OS, etc.) for analysis and identified the advantages and disadvantages of neoadjuvant immunotherapy for non-metastatic dMMR CRC (66). We aimed to standardize the dosage and treatment course and adopted subgroup analysis and sensitivity analysis to evaluate inconsistencies. When significant heterogeneity was observed, we used a random-effects model to combine studies with different designs or schemes, striving to minimize heterogeneity.

The cCR rate was 31.4% in our study. This was significantly higher than that of patients receiving neoadjuvant chemotherapy or pMMR patients with neoadjuvant immunotherapy. Cercek et al. (2022) reported that 12 patients had achieved cCR and no case had progression or recurrence during follow-up (6 to 25 months) (20). Pei et al. (2023) reported that all the patients in their study had no recurrence or metastasis for 335 days in non-metastatic dMMR/MSI-H CRC after neoadjuvant immunotherapy, while Xiao et al. (2023) found that there was no recurrence or metastasis for 2 years (34, 35). The NICHE-1 trial was the first phase II clinical study to investigate neoadjuvant immunotherapy in early-stage non-metastatic colon cancer. For dMMR/MSI-H patients, the trial demonstrated a major MPR rate of 95% (19/20) and pCRs rate of 60% (12/20). With a median follow-up of 25 months, none of the dMMR/MSI-H patients experienced recurrence, resulting in a 100% recurrence-free survival (RFS) rate (10). Neoadjuvant immunotherapy in dMMR/MSI-H CRC demonstrated remarkable pCRs rate and short-term survival advantages, but its conversion to long-term survival benefits still required higher-level evidence. Future efforts needed to focus on refinement of biomarkers, optimization of combination regimens, and long-term follow-up to validate the causal relationship between pathological responses and survival outcomes.

In dMMR/MSI-H tumors, although immunotherapy significantly improved the prognosis of some patients, approximately 20%–40% of patients exhibited primary or secondary resistance. These resistance mechanisms were complex, involving tumor-intrinsic characteristics, microenvironment regulation, and host immune status (67). Loss or mutation of HLA class I molecules and insufficient antigen diversity leads to antigen presentation defects, resulting in immune escape. Activation of oncogenic signaling pathways (Wnt/β-catenin pathway activation and IFN-γ signaling defects) and DNA methylation or chromatin remodeling contribute to tumor resistance (68, 69). Therefore, future research is needed to reveal resistance dynamics through multi-omics analysis (genomics, transcriptomics, and immunomics) and develop precise combination strategies to facilitate early identification of resistance and optimize treatment decisions.

### Limitations

There were several limitations in the meta-analysis. First, inconsistencies between the neoadjuvant immunotherapy drugs and regimens included in the article may affect the results. Second, limited clinical data for neoadjuvant immunotherapy for non-metastatic dMMR/MSI-H CRC could affect the results. Third, single-arm studies without a control group can often enroll highly selected patients (those with good performance status, normal organ function, and others), which limits the generalizability of the results to real-world populations and increases the risk of selection bias. Single-arm studies focus primarily on efficacy endpoints such as ORR, MPR, and pCR, and do not assess PFS and OS (overall survival), which had certain limitations and could affect the robustness of conclusions.

## Conclusion

Based on the above results, neoadjuvant immunotherapy was found to increase MPR and pCR rates for patients with non-metastatic dMMR/MSI-H CRC. Neoadjuvant immunotherapy did not increase the incidence of postoperative complications and irAEs, while neoadjuvant immune monotherapy can further reduce irAEs compared with dual immunotherapy combined with chemo(radio)therapy. For patients with locally advanced T4 stage dMMR/MSI-H CRC, neoadjuvant immunotherapy can still achieve good pCR rates. We expect that precise neoadjuvant immunotherapy drugs and regimens will be developed, which will increase MPR and pCR rates and other outcomes. furthermore, we also look forward to the emergence of more RCTs that can confirm the clinical effects of neoadjuvant immunotherapy for non-metastatic dMMR/MSI-H CRC. Neoadjuvant immunotherapy could be another treatment option for non-metastatic dMMR/MSI-H CRC, especially for locally advanced T4 stage dMMR/MSI-H CRC.

## Data Availability

The original contributions presented in the study are included in the article/**Supplementary Material**. Further inquiries can be directed to the corresponding author.
