# Supplementary material for: The neoadjuvant immunotherapy for non-metastatic mismatch repair-deficient colorectal cancer: a systematic review
Source: Front Immunol. 2025 May 1;16:1540751. doi: 10.3389/fimmu.2025.1540751 (PMC12078204; doi:10.3389/fimmu.2025.1540751)
Supplement: Supplementary file 1 [file DataSheet1.pdf]

National Institute for Health Research

UNIVERSITY of York
Centre for Reviews and Dissemination

# Systematic review

A list of fields that can be edited in an update can be found here

## 1. * Review title.

Give the title of the review in English

Systematic review of neoadjuvant immunotherapy for non-metastatic mismatch repair-deficient colorectal cancer

## 2. Original language title.

For reviews in languages other than English, give the title in the original language. This will be displayed with the English language title.

Systematic review of neoadjuvant immunotherapy for non-metastatic mismatch repair-deficient colorectal cancer

## 3. * Anticipated or actual start date.

Give the date the systematic review started or is expected to start.

01/09/2024

## 4. * Anticipated completion date.

Give the date by which the review is expected to be completed.

31/12/2024

## 5. * Stage of review at time of this submission.

**This field uses answers to initial screening questions. It cannot be edited until after registration.**

Tick the boxes to show which review tasks have been started and which have been completed.

Update this field each time any amendments are made to a published record.

The review has not yet started: No

| | | |
|---|---|---|
| Preliminary searches | Yes | No |
| Piloting of the study selection process | Yes | No |
| Formal screening of search results against eligibility criteria | No | No |
| Data extraction | No | No |
| Risk of bias (quality) assessment | No | No |
| Data analysis | No | No |

Provide any other relevant information about the stage of the review here.

Part of the work has already started, and there are still some unfinished tasks

Part of the work has already started, and there are still some unfinished tasks

## 6. * Named contact.

The named contact is the guarantor for the accuracy of the information in the register record. This may be any member of the review team.

Xin Liu

## Email salutation (e.g. "Dr Smith" or "Joanne") for correspondence:

Mr Liu

## 7. * Named contact email.

Give the electronic email address of the named contact.

liuxin5626855@sina.com

## 8. Named contact address

Give the full institutional/organisational postal address for the named contact.

Cancer hospital of China medical university, Liaoning cancer hospital and institute\nshenyang, xiao he yan road, No.44, Liaoning Province CHINA

## 9. Named contact phone number.

Give the telephone number for the named contact, including international dialling code.

8618900918981

## 10. * Organisational affiliation of the review.

Full title of the organisational affiliations for this review and website address if available. This field may be completed as 'None' if the review is not affiliated to any organisation.

Cancer Hospital of China Medical University. Liaoning Cancer Hospital and Institute

## Organisation web address:

## 11. * Review team members and their organisational affiliations.

Give the personal details and the organisational affiliations of each member of the review team. Affiliation refers to groups or organisations to which review team members belong. **NOTE: email and country now MUST be entered for each person, unless you are amending a published record. PLEASE USE AN INSTITUTIONAL EMAIL ADDRESS IF POSSIBLE.**

Mr Xin Liu. Cancer Hospital of China Medical University. Liaoning Cancer Hospital and Institute
Mrs Hongxia Cui. Department of Pharmacy, Cancer Hospital of China Medical University. Liaoning Cancer Hospital & Institute
Mr XiaoQuan Yang. Department of general surgery, Cancer Hospital of China Medical University. Liaoning Cancer Hospital & Institute
Mr Guangyue Zhao. Department of Colorectal surgery, Cancer Hospital of China Medical University. Liaoning Cancer Hospital & Institute
Mr Fengjian Wang. Department of Colorectal surgery, Cancer Hospital of China Medical University. Liaoning Cancer Hospital & Institute

## 12. * Funding sources/sponsors.

Details of the individuals, organizations, groups, companies or other legal entities who have funded or sponsored the review.

no

## Grant number(s)

State the funder, grant or award number and the date of award

0

## 13. * Conflicts of interest.

List actual or perceived conflicts of interest (financial or academic).

None

## 14. Collaborators.

Give the name and affiliation of any individuals or organisations who are working on the review but who are not listed as review team members. **NOTE: email and country must be completed for each person, unless you are amending a published record.**

## 15. * Review question.

State the review question(s) clearly and precisely. It may be appropriate to break very broad questions down into a series of related more specific questions. Questions may be framed or refined using PI(E)COS or similar where relevant.

Guidelines have recommend pembrolizumab for the first-line treatment for metastatic dMMR/MSI-H CRC (11). Although some studies have confirmed the efficacy of neoadjuvant immunotherapy for locally advanced dMMR/MSI-H CRC, there are no relevant guidelines recommending whether neoadjuvant immunotherapy could be used in non-metastatic dMMR/MSI-H colorectal cancer(12). Therefore, we collected relevant articles of neoadjuvant immunotherapy for non-metastatic dMMR/MSI-H CRC and tried to explain the safety and efficacy of neoadjuvant immunotherapy for non-metastatic dMMR/MSI-H CRC.

## 16. * Searches.

State the sources that will be searched (e.g. Medline). Give the search dates, and any restrictions (e.g. language or publication date). Do NOT enter the full search strategy (it may be provided as a link or attachment below.)

We obtained relevant articles by consulting relevant databases ((Embase, PubMed, Cochrane Library, CNKI (China National Knowledge Infrastructure) and Wanfang databases)). We finished the literature search on September 2024. The search terms were “colorectal cancer”, “neoadjuvant immunotherapy”and “mismatch repair-deficient”.

## 17. URL to search strategy.

Upload a file with your search strategy, or an example of a search strategy for a specific database, (including the keywords) in pdf or word format. In doing so you are consenting to the file being made publicly accessible. Or provide a URL or link to the strategy. Do NOT provide links to your search **results**.

("colorectal neoplasms"[MeSH Terms] OR ("colorectal"[All Fields] AND "neoplasms"[All Fields]) OR "colorectal neoplasms"[All Fields] OR ("colorectal"[All Fields] AND "cancer"[All Fields]) OR "colorectal cancer"[All Fields]) AND (("neoadjuvancy"[All Fields] OR "neoadjuvant therapy"[MeSH Terms] OR ("neoadjuvant"[All Fields] AND "therapy"[All Fields]) OR "neoadjuvant therapy"[All Fields] OR "neoadjuvant"[All Fields] OR "neoadjuvants"[All Fields] OR "neoadjuvent"[All Fields]) AND ("immunotherapy"[MeSH Terms] OR "immunotherapy"[All Fields] OR "immunotherapies"[All Fields] OR "immunotherapy s"[All Fields])) AND (("mismatch"[All Fields] OR "mismatched"[All Fields] OR "mismatches"[All Fields] OR "mismatching"[All Fields]) AND "repair-deficient"[All Fields])

Alternatively, upload your search strategy to CRD in pdf format. Please note that by doing so you are consenting to the file being made publicly accessible.

Do not make this file publicly available until the review is complete

## 18. * Condition or domain being studied.

Give a short description of the disease, condition or healthcare domain being studied in your systematic review.

The high incidence rate of colorectal cancer (CRC) has seriously threatened human health (1). CRC is classified into mismatch repair-deficient (dMMR) CRC and mismatch repair–proficient (pMMR) CRC based on the absence of MMR protein expression (2). DMMR CRC accounts for approximately 15% of non-metastatic CRC patients (3, 4). Neoadjuvant therapy is one of the important treatment modes for CRC, especially for rectal cancer (5, 6). FOxTROT study reported that most dMMR colon cancer patients had little or no response with neoadjuvant chemotherapy (FOLFOX or CAPOX) (7). Some relevant retrospective studies showed that dMMR rectal cancer had disease progression with neoadjuvant chemotherapy (fluorouracil plus oxaliplatin), but there was no disease progression in the pMMR group with neoadjuvant chemotherapy (8).

## 19. * Participants/population.

Specify the participants or populations being studied in the review. The preferred format includes details of both inclusion and exclusion criteria.

non-metastatic mismatch repair-deficient colorectal cancer patients

## 20. * Intervention(s), exposure(s).

Give full and clear descriptions or definitions of the interventions or the exposures to be reviewed. The preferred format includes details of both inclusion and exclusion criteria.

right-side group

## 21. * Comparator(s)/control.

Where relevant, give details of the alternatives against which the intervention/exposure will be compared (e.g. another intervention or a non-exposed control group). The preferred format includes details of both inclusion and exclusion criteria.

left-side group

## 22. * Types of study to be included.

Give details of the study designs (e.g. RCT) that are eligible for inclusion in the review. The preferred format includes both inclusion and exclusion criteria. If there are no restrictions on the types of study, this should be stated.

prospective study, retrospective study, single-arm study, cohort study and RCTs

## 23. Context.

Give summary details of the setting or other relevant characteristics, which help define the inclusion or exclusion criteria.

## 24. * Main outcome(s).

Give the pre-specified main (most important) outcomes of the review, including details of how the outcome is defined and measured and when these measurement are made, if these are part of the review inclusion criteria.

MPR, pCRs

## Measures of effect

Please specify the effect measure(s) for you main outcome(s) e.g. relative risks, odds ratios, risk difference, and/or 'number needed to treat.

## 25. * Additional outcome(s).

List the pre-specified additional outcomes of the review, with a similar level of detail to that required for main outcomes. Where there are no additional outcomes please state 'None' or 'Not applicable' as appropriate to the review

postoperative complications, immune-related adverse events (irAEs), T4-pCRs and ORR

## Measures of effect

Please specify the effect measure(s) for you additional outcome(s) e.g. relative risks, odds ratios, risk difference, and/or 'number needed to treat.

## 26. * Data extraction (selection and coding).

Describe how studies will be selected for inclusion. State what data will be extracted or obtained. State how this will be done and recorded.

Two reviewers (GYZ and FJW) searched the relevant literatures and sorted the useful clinical data independently with the help of the revised version of MINORS (methodological index for non-randomized studies) (14). The revised version of MINORS was used for the quality assessment of observational or non-randomized studies(15). The third reviewer (LZ) resolved the inconsistencies between the above two authors.

## 27. * Risk of bias (quality) assessment.

State which characteristics of the studies will be assessed and/or any formal risk of bias/quality assessment tools that will be used.

Stata 11.0 and RevMan 5.0 software was used to analyze the dichotomous data, and it was evaluated by relative risks (ORs or RRs) with 95% confidence intervals.

## 28. * Strategy for data synthesis.

Describe the methods you plan to use to synthesise data. This **must not be generic text** but should be **specific to your review** and describe how the proposed approach will be applied to your data. If meta-analysis is planned, describe the models to be used, methods to explore statistical heterogeneity, and software package to be used.

Stata 11.0 and RevMan 5.0 software was used to analyze the dichotomous data, and it was evaluated by

relative risks (ORs or RRs) with 95% confidence intervals. Random effects models and fixed effects model were used to analyse the data with huge heterogeneity (I²?50%) and for little heterogeneity (I²50%) respectively. Publication bias was assessed by the funnel plots.

## 29. * Analysis of subgroups or subsets.

State any planned investigation of ‘subgroups’. Be clear and specific about which type of study or participant will be included in each group or covariate investigated. State the planned analytic approach.

Secondary outcomes (subgroup analysis): pCRs (T1-T3 vs T4a-T4b), pCRs (right-side vs left-side), pCRs

(colon cancer vs rectal cancer) and pCRs (mismatch repair-deficient vs [illegible]) [illegible] The population included non-metastatic [illegible] colorectal cancer patients. The outcomes

included pCRs.

## 30. * Type and method of review.

Select the type of review, review method and health area from the lists below.

### Type of review

Cost effectiveness

No

Diagnostic

No

Epidemiologic

No

Individual patient data (IPD) meta-analysis

No

Intervention

No

Living systematic review

No

Meta-analysis

No

Methodology

No

Narrative synthesis

No

Network meta-analysis

No

Pre-clinical

No

Prevention

No

Prognostic

No

Prospective meta-analysis (PMA)

No

Review of reviews

No

Service delivery

No

Synthesis of qualitative studies

No

Systematic review

Yes

Other

No

## Health area of the review

Alcohol/substance misuse/abuse

No

Blood and immune system

No

Cancer

Yes

Cardiovascular

No

Care of the elderly

No

Child health

No

Complementary therapies

No

COVID-19

No

Crime and justice

No

Dental

No

Digestive system

No

Ear, nose and throat

No

Education

No

Endocrine and metabolic disorders

No

Eye disorders

No

General interest

No

Genetics

No

Health inequalities/health equity

No

Infections and infestations

No

International development

No

Mental health and behavioural conditions

No

Musculoskeletal

No

Neurological

No

Nursing

No

Obstetrics and gynaecology

No

Oral health

No

Palliative care

No

Perioperative care

No

Physiotherapy

No

Pregnancy and childbirth

No

Public health (including social determinants of health)

No

Rehabilitation

No

Respiratory disorders

No

Service delivery

No

Skin disorders

No

Social care

No

Surgery

No

Tropical Medicine

No

Urological

No

Wounds, injuries and accidents

No

Violence and abuse

No

## 31. Language.

Select each language individually to add it to the list below, use the bin icon  to remove any added in error.
English

There is not an English language summary

## 32. * Country.

Select the country in which the review is being carried out. For multi-national collaborations select all the countries involved.

China

## 33. Other registration details.

Name any other organisation where the systematic review title or protocol is registered (e.g. Campbell, or The Joanna Briggs Institute) together with any unique identification number assigned by them. If extracted data will be stored and made available through a repository such as the Systematic Review Data Repository (SRDR), details and a link should be included here. If none, leave blank.

no

## 34. Reference and/or URL for published protocol.

If the protocol for this review is published provide details (authors, title and journal details, preferably in Vancouver format)

no

Add web link to the published protocol.

Or, upload your published protocol here in pdf format. Note that the upload will be publicly accessible.

No I do not make this file publicly available until the review is complete

Please note that the information required in the PROSPERO registration form must be completed in full even if access to a protocol is given.

## 35. Dissemination plans.

Do you intend to publish the review on completion?

No

Give brief details of plans for communicating review findings.?

## 36. Keywords.

Give words or phrases that best describe the review. Separate keywords with a semicolon or new line. Keywords help PROSPERO users find your review (keywords do not appear in the public record but are included in searches). Be as specific and precise as possible. Avoid acronyms and abbreviations unless these are in wide use.

neoadjuvant immunotherapy, non-metastatic colorectal cancer, mismatch repair-deficient, meta-analysis

## 37. Details of any existing review of the same topic by the same authors.

If you are registering an update of an existing review give details of the earlier versions and include a full bibliographic reference, if available.

## 38. * Current review status.

Update review status when the review is completed and when it is published.New registrations must be ongoing so this field is not editable for initial submission.

Please provide anticipated publication date

Review_Ongoing

## 39. Any additional information.

Provide any other information relevant to the registration of this review.

no

## 40. Details of final report/publication(s) or preprints if available.

Leave empty until publication details are available OR you have a link to a preprint (NOTE: this field is not editable for initial submission). List authors, title and journal details preferably in Vancouver format.

Give the link to the published review or preprint.
