# Supplementary material for: The neoadjuvant immunotherapy for non-metastatic mismatch repair-deficient colorectal cancer: a systematic review
Source: Front Immunol. 2025 May 1;16:1540751. doi: 10.3389/fimmu.2025.1540751 (PMC12078204; doi:10.3389/fimmu.2025.1540751)
Supplement: Supplementary file 3 [file DataSheet3.pdf]

Supplementary material 3: clinical stage, T stage and N stage of the included articles

Study	Clinical stage		Clinical T stage			Clinical N stage	
	II	III	T1-T2	T3	T4	N0	N+
Bando H 2022	2	3	0	5	0	3	2
Cercek A 2022	NR	NR	4	9	3	1	15
Chalabi M 2024	NR	NR	17	24	74	38	77
Chen G 2023	NR	NR	2	10	5	3	14
de Gooyer PGM 2024	NR	NR	1	18	40	22	37
Deng Z 2024	0	17	0	7	10	0	17
Han K 2023	NR	NR	NR	NR	NR	NR	NR
Hu H 2022	4	32	0	6	30	4	32
Kothari A 2022	1	8	0	3	6	1	8
Li YJ 2023	1	18	0	10	9	1	18
Li YJ 2024	1	16	0	7	10	1	16
Liu DX 2024	0	4	0	3	1	0	4
Liu ZX 2022	NR	NR	NR	NR	NR	NR	NR
Ludford K 2023	NR	NR	NR	NR	NR	NR	NR
Pan T 2024	2	8	0	8	2	2	8
Pei F 2023	3	8	0	6	5	3	8
Xiao BY 2023	NR	NR	3	22	48	7	66
Xie Y 2023	0	13	NR	NR	NR	NR	NR
Yang R 2023	6	14	0	7	13	6	14
Yu JH 2024	NR	NR	0	15	37	2	50
Zhang X 2022	4	28	0	6	26	4	28

Notes: dMMR: mismatch repair-deficient; MSI-H: microsatellite instability-high; pCR: pathological complete response; MPR: major pathological response; ORR: objective response rate; cCR: complete clinical response; CRT: chemoradiotherapy; irAEs: immune-related adverse events; RCS: Retrospective clinical study; PCS: Prospective clinical study; NR: no record.

The orders of additional information were range, standard deviation, percentage or NR (if not reported).
