# Supplementary material for: The neoadjuvant immunotherapy for non-metastatic mismatch repair-deficient colorectal cancer: a systematic review
Source: Front Immunol. 2025 May 1;16:1540751. doi: 10.3389/fimmu.2025.1540751 (PMC12078204; doi:10.3389/fimmu.2025.1540751)
Supplement: Supplementary file 4 [file DataSheet4.pdf]

Supplementary material 4: the details of neoadjuvant immunotherapy plan

Study	**Neoadjuvant immunotherapy**
Bando H 2022	The study treatments included five cycles of nivolumab monotherapy (240 mg every 2 weeks) and subsequent radical surgery using a sphincter-sparing procedure or abdominoperineal resection with TME, after CRT with capecitabine and radiation to a dose of 50.4 Gy, in 28 fractions. The period between the end of CRT and radical surgery was planned for 12 weeks.
Cercek A 2022	Neoadjuvant dostarlimab administered intravenously at a dose of 500 mg every 3 weeks for 6 months (nine cycles) was to be followed by standard radiation therapy (total dose of 5040 cGy given in 28 fractions), with concurrent administration of capecitabine at standard doses and then total mesorectal excision.
Chalabi M 2024	Patients received two doses of nivolumab at a dose of 3 mg per kilogram of body weight, with the first dose administered on day 1 and the second on day 15, and one dose of ipilimumab at a dose of 1 mg per kilogram on day 1. Surgery was scheduled to be performed at one of six participating centers in the Netherlands within 6 weeks after study enrollment.
Chen G 2023	Patients in this trial received sintilimab at a fixed dose of 200 mg by intravenous infusion on day 1 every 3 weeks. After completion of two initial cycles of treatment with sintilimab, response was evaluated.
de Gooyer PGM 2024	Patients were treated with a short regimen of two cycles of nivolumab (480 mg) plus relatlimab (480 mg) on day 1 and day 29, followed by surgery within 8 weeks of enrollment
Deng Z 2024	Nivolumab was administered 3mg/kg for 2 cycles and ipilimab 1mg/kg for 1 cycle according to NICHE-2 study protocol. Patients using single-agent immunotherapy received 200mg intravenous infusion every three weeks until the tumor regressed to undergo radical resection.
Han K 2023	Patients received 4 cycles of anti-PD1 monotherapy (pembrolizumab, 200 mg, q3w; or nivolumab, 3 mg/kg, q2w), and surgery would be done in about 4-6 weeks after the neadjuvant therapy.
Hu H 2022	In both treatment groups, toripalimab 3 mg/kg (Junshi BioSciences, Shanghai, China) was given intravenously over 30 min on day 1 of each 14-day cycle, for six cycles, before surgical resection. Surgery was planned for within 4 weeks after the last neoadjuvant toripalimab dose. Dose modifications were not permitted, but toripalimab or celecoxib, or both, could be interrupted or delayed for a maximum of 2 weeks.
Kothari A 2022	Four patients received chemotherapy before initiation of CPI treatment, with a mean of four cycles of treatment. The median number of immunotherapy treatment doses was 5 (range 1–16).<br>The median interval from initiation of CPI therapy to surgery was 5 months, and the interval between the last dose of CPI and surgery was 48 days.
Li YJ 2023	All patients received PD1 blockade (PD1 blockade 200 mg intravenously over 30 min on day 1 of each 21-d cycle) preoperative immunotherapy with or without CapOx chemotherapy (oxaliplatin 130 mg/m^2 on day 1 and capecitabine 1000mg/m^2 twice daily on d 1–14, repeated every 3 wk).
Li YJ 2024	All patients received at least 1 dose of PD-1 blockade, usually 200mg intravenously over 30 minutes on day 1, every 21 days. Effectiveness evaluation was performed every 1 or 3 cycles, at the discretion of the attending physician, and included a CT scan and/or MRI, PET/CT, endoscopy, and tumor markers.
Liu DX 2024	Pembrolizumab 200, 240 or 250mg intravenously, 2-4 cycles; nivolumab 250mg intravenously, 12 cycles.
Liu ZX 2022	Patients were treated with PD-1 single agent (2 cycles)±long-course radiotherapy for rectal cancer; PD-1 single agent was used for colon cancer.
Ludford K 2023	The planned treatment course was pembrolizumab 200 mg intravenously once every 3 weeks for eight treatments followed by surgical resection.
Pan T 2024	10 patients were treated with one dose of ipilimumab (1mg/kg) and two doses of nivolumab (3mg/kg), and one patient was treated with one dose of ipilimumab (1mg/kg) and two doses of nivolumab (3mg/kg) with two cycles.
Pei F 2023	All patients received neoadjuvant immunotherapy in the form of 200 mg sintilimab injection intravenously on day 1 of each 3-week cycle, for a total of 6 injections. Radical laparoscopic resection for all patients was scheduled to be completed within 14 to 30 days after the end of the last neoadjuvant immunotherapy treatment.
Xiao BY	Patients received ≥2 doses of PD-1 inhibitors (including pembrolizumab, nivolumab, sintilimab, toripalimab, camrelizumab, and tislelizumab).

2023	Neoadjuvant radiation and chemotherapy(FOLFIRI, FOLFOX, FOLFOXIR and XELOX) was used for several patients.
Xie Y 2023	4 cycles of pembrolizumab, 200 mg every 3 weeks, 6 cycles of toripalimab, 3 mg/kg every 2 weeks, 6 cycles of sintilimab, 200 mg every 3 weeks
Yang R 2023	Each patient received 200 mg of a PD-1 inhibitor by intravenous infusion every 3 weeks until tumor regression to feasible R0 resection, cCR, or near-cCR. There were no limits on the type of PD-1 inhibitors, and these included pembrolizumab, sintilimab, and tislelizumab.
Yu JH 2024	Patients were given 200 mg intravenous camrelizumab on day 1 and 250 mg oral apatinib on days 1–14 every 3 weeks.
Zhang X 2022	All 32 patients who met the inclusion and exclusion criteria received nIT with single-agent PD-1 inhibitor, of which 4 patients used pembrolizumab, 9 patients used sintilimab and 19 patients treated with tiselizumab. Within the first day of each treatment cycle, they received nIT with intravenous drip of 200mg PD-1 inhibitor. The duration of each cycle of treatment was 3 weeks, regardless of how many courses of treatment were used (200 mg IV Q3W). Before using nIT, regardless of whether you have received nCT or nCRT. All patients underwent radical resection or W&W strategy. Of the 29 patients who underwent surgery, 22 received adjuvant immunotherapy with PD-1 blockade.

Notes: dMMR: deficient mismatch repair; MSI-H: microsatellite instability-high; MSI-L: microsatellite instability-low; pMMR: proficient mismatch repair; MSS: microsatellite stable; CRM: circumferential resection margin; EMVI: extramural venous invasion; pCR: pathological complete response; MPR: major pathological response; ORR: objective response rate; cCR: complete clinical response; SBRT: stereotactic body radiation therapy; IMRT: implementation of intensity-modulated radiotherapy; NR: no record.

The orders of additional information were range, standard deviation, percentage or NR (if not reported).
