# Supplementary material for: The neoadjuvant immunotherapy for non-metastatic mismatch repair-deficient colorectal cancer: a systematic review
Source: Front Immunol. 2025 May 1;16:1540751. doi: 10.3389/fimmu.2025.1540751 (PMC12078204; doi:10.3389/fimmu.2025.1540751)
Supplement: Supplementary file 5 [file DataSheet5.pdf]

Supplementary material 5: MINORS score of the included articles

Criterion	Study									
	Bando H 2022	Cercek A 2022	Chalabi M 2024	Chen G 2023	de Gooyer PGM 2024	Deng Z 2024	Han K 2023	Hu H 2022	Kothari A 2022	Li YJ 2023
1. A stated aim of the study	2	2	2	2	2	2	2	2	2	2
2. Inclusion of consecutive patients	2	2	2	2	2	2	2	2	2	2
3. Prospective collection of data	0	2	0	0	0	0	0	2	0	0
4. Endpoint appropriate to the study aim	2	2	2	2	2	2	2	2	2	2
5. Unbiased evaluation of endpoints	2	2	2	2	2	2	2	2	2	2
6. Follow-up period appropriate to the major endpoint	2	2	2	2	2	2	2	2	2	2
7. Loss to follow up not exceeding 5%	2	2	2	2	2	2	2	2	2	2
8. Prospective calculation of the study size:	0	0	2	2	0	0	0	0	0	0
Sum	12	14	14	14	12	12	12	14	12	12

Criterion	Study										
	Li YJ 2024	Liu DX 2024	Liu ZX 2022	Ludford K 2023	Pan T 2024	Pei F 2023	Xiao BY 2023	Xie Y 2023	Yang R 2023	Yu JH 2024	Zhang X 2022
1. A stated aim of the study	2	2	2	2	2	2	2	2	2	2	2
2. Inclusion of consecutive patients	2	2	2	2	2	2	2	2	2	2	2
3. Prospective collection of data	0	0	0	0	0	0	0	0	0	0	0
4. Endpoint appropriate to the study aim	2	2	2	2	2	2	2	2	2	2	2
5. Unbiased evaluation of endpoints	2	2	2	2	2	2	2	2	2	2	2
6. Follow-up period appropriate to the major endpoint	2	2	2	2	2	2	2	2	2	2	2
7. Loss to follow up not exceeding 5%	2	2	2	2	2	2	2	2	2	2	2
8. Prospective calculation of the study size:	0	0	0	0	0	0	0	0	0	2	0
Sum	12	12	12	12	12	12	12	12	12	14	12

Notes: dMMR: mismatch repair-deficient; MSI-H: microsatellite instability-high; pCR: pathological complete response; MPR: major pathological response; ORR: objective response rate; cCR: complete clinical response; CRT: chemoradiotherapy; irAEs: immune-related adverse events; RCS: Retrospective clinical study; PCS: Prospective clinical study; NR: no record.

The orders of additional information were range, standard deviation, percentage or NR (if not reported).
